# Impacts of two types of ENSO on tropical cyclone tracks affecting South Korea: A clustering-based approach

**DOI:** 10.1016/j.isci.2025.113824

**Published:** 2025-10-21

**Authors:** Han-Kyoung Kim, Jong-Yeon Park, Jung-Eun Chu

**Affiliations:** 1 Department of Earth and Environmental Sciences & Earth Environmental System Research Center, Jeonbuk National University; 2 Department of Environment and Energy, Jeonbuk National University, Jeonju, South Korea; 3 School of Energy and Environment, City University of Hong Kong, Hong Kong, China

**Keywords:** earth sciences, atmospheric science, climatology

## Abstract

The impacts of different ENSO types on tropical cyclone (TC) tracks affecting South Korea remain less understood, despite extensive studies on their influence on overall western North Pacific (WNP) TC activity. Using a self-organizing map, we classify TC tracks entering the South Korean emergency zone into two clusters. Cluster 1 (C1) TCs form in the Philippine Sea and travel northward. During eastern Pacific La Niña events, a strengthened Walker circulation and a Gill-type response produce southerly wind anomalies along C1 pathway, increasing C1 frequency. Cluster 2 (C2) TCs originate in the southeastern WNP and follow recurving tracks. During central Pacific El Niño events, a Gill-type response induces a cyclonic circulation anomaly over the southeastern WNP, which enhances TC genesis and shifts the subtropical high eastward, increasing C2 frequency. These results reveal distinct dynamic processes through which different ENSO types modulate TC behavior, improving forecast skill for TCs affecting South Korea.

## Highlights

• TC tracks affecting South Korea are classified into two clusters using SOM
• EP and CP ENSO exert distinct influences on the clustered TC tracks
• Findings improve understanding and prediction of ENSO-TC relationships over Korea

## Graphical abstract

**Figure:**
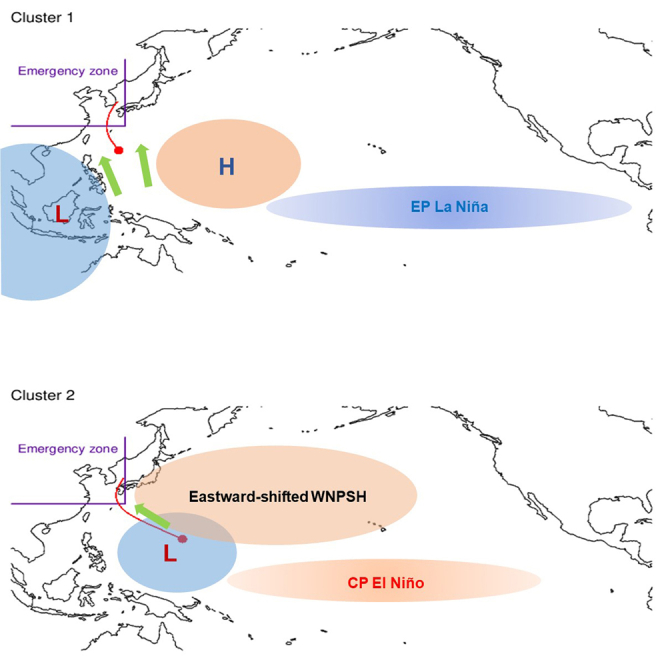

## Introduction

Tropical cyclones (TCs) are among the most destructive weather events, yet they play a crucial role in regulating Earth’s energy balance between the equator and higher latitudes.^1^^,^^2^^,^^3^ In particular, the western North Pacific (WNP) is one of the most active TC basins, accounting for approximately one-third of global TC occurrences.^4^^,^^5^^,^^6^

South Korea, situated in East Asia, experiences an average of approximately four TCs annually originating from the WNP. In years of high TC activity, the country faces threats to livelihoods due to TC-induced strong winds and heavy rainfall, resulting in substantial socio-economic losses. Conversely, in years of low TC activity, South Korea is vulnerable to challenges such as water resource shortages. Over the past two decades, TC-related damages have accounted for an average of 32% of the total losses caused by meteorological hazards.^7^ Therefore, understanding the underlying mechanisms driving the interannual variability of TCs affecting South Korea is crucial for mitigating their adverse effects.

The El Niño-southern oscillation (ENSO), a well-known coupled atmosphere-ocean phenomenon, is the primary climate driver influencing TC activity over the WNP on the interannual timescale.^5^^,^^8^^,^^9^^,^^10^^,^^11^^,^^12^^,^^13^ Notably, previous studies have shown that ENSO does not exert uniform impacts across the entire WNP, but instead has regionally distinct effects.^5^^,^^9^ Wang and Chan,^9^ using the Niño-3.4 index, demonstrated that during El Niño years, TC genesis increases in the southeastern WNP while decreasing in the northwestern WNP. However, the total number of TCs across the WNP does not exhibit a significant relationship with the Niño-3.4 index. They also observed a significant increase in northward-recurving TCs originating in the southeastern WNP during El Niño years, attributed to the deepening of the East Asian trough and the retreat of subtropical high.

Different types of ENSO events further influence WNP TC activity in distinct ways.^4^^,^^5^^,^^11^^,^^14^ Kim and Seo^5^ found that central Pacific (CP) El Niño events induce a cyclone Rossby wave response northwest of the sea surface temperature (SST) forcing, leading to increased TC genesis in the eastern WNP. In contrast, eastern Pacific (EP) El Niño events weaken the Walker circulation, resulting in decreased TC genesis in the western WNP.

These findings suggest that ENSO and its diversity (i.e., different types of ENSO events) may influence TCs affecting South Korea, as the impacts of TCs are largely determined by their genesis location and track. In this context, previous studies have attempted to establish a relationship between ENSO and TCs affecting South Korea.^15^^,^^16^ However, these studies consistently found no significant correlation between TC frequency affecting South Korea and the Niño-3.4 index.^15^^,^^16^ Nonetheless, Zhang et al.^14^ reported a higher probability of TCs affecting Korea and Japan during boreal summer CP El Niño years, although no significant relationship was found between TC frequency in East Asia and EP El Niño and La Niña years during boreal summer.

**Figure 1:**
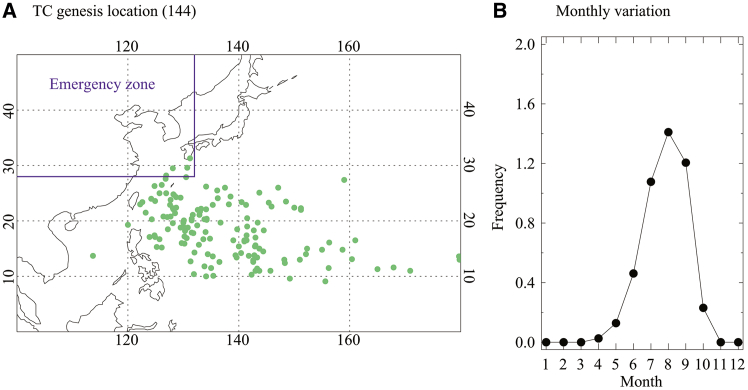
Spatiotemporal characteristics of TCs affecting South Korea (A) Genesis locations and (B) monthly variation of 144 TCs entering the South Korean emergency zone (west of 132°E and north of 28° N).

The genesis locations of TCs affecting South Korea during the peak season (July to September, hereafter JAS; Figure 1B) from 1982 to 2020 are widely distributed across the entire WNP (Figure 1A). Due to the existence of different types of ENSO and their regionally distinct influences on TC activity in the WNP, establishing a robust relationship between the total frequency of TCs affecting South Korea and ENSO remains challenging. In this context, clustering analysis of TC trajectories, including their genesis locations, offers a useful alternative for identifying distinct TC patterns that affect South Korea and are related to ENSO. In particular, given the nonlinear nature of TC tracks, the use of a self-organizing map (SOM), which is an unsupervised artificial neural network well-suited for recognizing nonlinear patterns,^5^^,^^17^^,^^18^ is appropriate for classifying TC tracks that impact South Korea and for investigating the ENSO-related dynamical mechanisms associated with each cluster.

## Results

### Optimal cluster number and characteristics of the SOM cluster patterns

**Figure 2:**
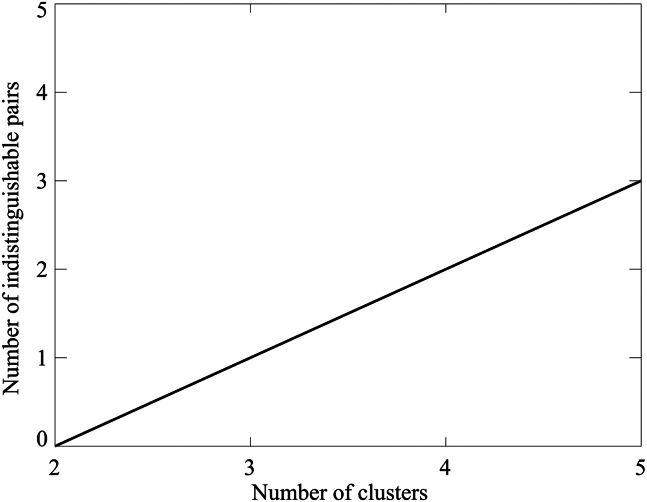
Result of the FDR test Number of statistically indistinguishable SOM cluster pairs as a function of cluster number.

To determine the optimal number of clusters for TC tracks entering the emergency zone in South Korea, SOM clustering is performed with the number of clusters varying from two to five. The clustering results are then evaluated using false discovery rate (FDR). Figure 2 illustrates the FDR outcome, showing the number of statistically indistinguishable cluster pairs as a function of the cluster number. When there are only two clusters, the clusters are statistically distinguishable. However, when the number of clusters is three or more, at least one pair of clusters becomes statistically indistinguishable. Therefore, the maximum number of statistically distinguishable clusters is determined to be two.

**Figure 3:**
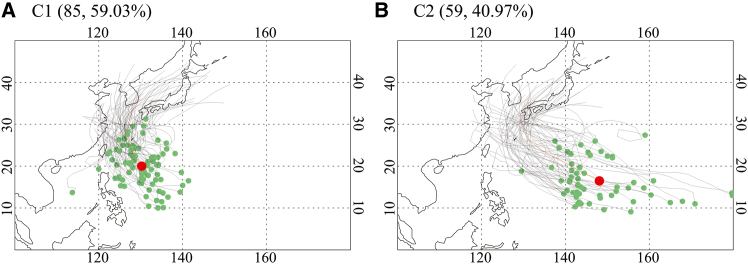
Two TC clusters identified using SOM (A) C1 and (B) C2. Individual TC genesis locations and tracks are indicated by green dots and black lines, respectively. The red dot and line denote the mean genesis position and track for each cluster pattern. The number of TCs and their percentage for each cluster are shown in the parentheses.

Cluster 1 (C1) accounts for 59.03% (85/144) of the total TCs affecting South Korea and primarily develops over the Philippine Sea (Figure 3A). This cluster is characterized by relatively weak intensity, with a minimum SLP of 961.17 hPa, a lifetime maximum wind speed (LMWS) of 36.19 m s^−1^, and a short duration of 5.32 days (Table 1). These characteristics are closely related to the mean genesis location of C1, which is situated at 130.28°E, 20.00°N (Table 1 and red dot in Figure 3A). Since C1 TCs tend to form near the emergency zone in South Korea, they have limited thermal energy supply from the ocean, contributing to their weaker intensity and shorter duration.

**Table 1:** Mean physical properties of the two TC SOM clusters

	Duration	Minimum SLP	LMWS	Genesis location
C1	5.32	961.17	36.19	130.28, 20.00
C2	8.58	935.92	45.99	148.10, 16.47

Mean duration (day), minimum SLP (hPa), LMWS (m s^−1^), and genesis location (longitude, latitude; °).

Cluster 2 (C2) consists of TCs that form further southeast in the WNP, with a mean genesis location at 148.10°E, 16.47°N (Table 1 and red dot in Figure 3B). Compared to C1, the mean genesis location of C2 is shifted approximately 18° eastward. Consequently, C2 TCs remain over warm ocean waters for a longer period before entering the emergency zone in South Korea. As a result, C2 is characterized by a longer duration of 8.58 days and greater intensity, with a minimum SLP of 935.92 hPa and an LMWS of 45.99 m s^−1^ (Table 1). About 40.97% (59/144) of all TCs belong to this cluster, making it less frequent than C1. The distinct characteristics of these two cluster patterns, particularly their genesis locations and durations, suggest that large-scale climate variability, such as ENSO, may influence their formation and trajectory. This possibility will be further examined in the next section.

### Impacts of two types of ENSO on the two SOM clusters

**Figure 4:**
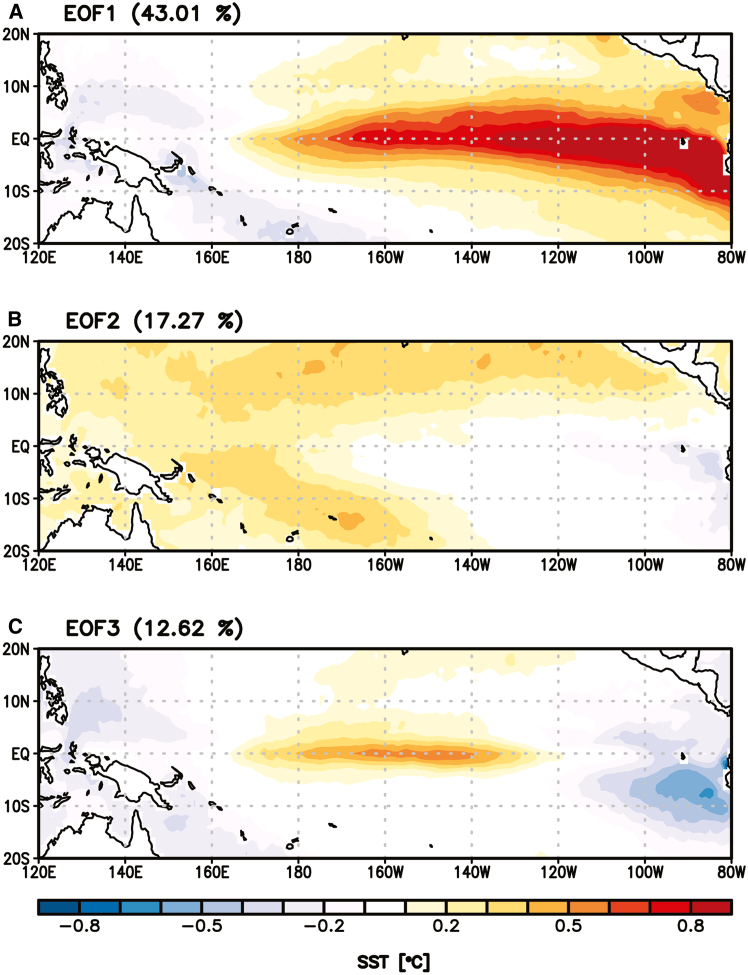
The three leading EOF modes of JAS mean SST anomalies over the tropical Pacific (120°E−80°W, 20°S-20°N) (A) Mode corresponding to EP El Niño, (B) cold tongue mode, and (C) CP El Niño. The percentage of variance explained by each EOF mode is indicated in the parentheses.

To investigate the relationship between each cluster pattern and ENSO-related variation, we first perform an empirical orthogonal function (EOF) analysis on the JAS mean SST anomalies over the tropical Pacific (120°E−80°W, 20°S-20°N) to extract the dominant modes associated with the EP and CP ENSO events. The first EOF (EOF1) mode represents the well-known EP El Niño pattern, explaining 43.01% of the tropical Pacific SST variability^19^^,^^20^ (Figure 4A). The second EOF mode is characterized by cooling in the EP region and warming in the remainder of the tropical Pacific, commonly referred to as the cold tongue mode^21^^,^^22^^,^^23^^,^^24^ (Figure 4B). This mode exhibits a dominant trend likely driven by ocean dynamic processes responding to global warming.^21^ The third EOF (EOF3) mode, accounting for 12.62% of the variability, captures the CP El Niño pattern (Figure 4C), which is also recognized as El Niño Modoki.^19^ Based on these spatial patterns, the first principal component (PC1) and third PC (PC3) are used as indices representing the EP ENSO and CP ENSO, respectively.

The results reveal that the interannual frequency of C1 is significantly negatively correlated with PC1 (r = −0.43, *p* < 0.01), whereas PC3 shows a significant positive correlation with interannual frequency of C2 (r = 0.38, *p* < 0.05). It is worth noting that the correlation coefficients between the total frequency of TCs affecting South Korea and PC1 and PC3 are −0.09 and 0.21, respectively, neither of which reaches statistical significance without clustering. These results align with several previous studies^15^^,^^16^ and suggest that clustering analysis is a valuable approach for uncovering hidden signals that may be obscured in the raw dataset.

To further explore the statistical and dynamical relationships between C1 and EP ENSO, we divide PC1 into warm and cold phases using its standard deviation thresholds of 0.75 and −0.75, respectively. The selected warm (cold) PC1 phase corresponds to 6 (9) years (Table 2). As shown in Table 3, the warm PC1 phase is associated with a statistically significant reduction in the frequency of TCs affecting South Korea for C1 (0.83 years^−1^, *p* < 0.1), whereas it increases significantly to 3.33 years^−1^ (*p* < 0.05) in the cold PC1 phase.

**Table 2:** Selected warm and cold years for PC1 and PC3

Warm PC1	Cold PC1	Warm PC3	Cold PC3
**1982** 1983 1987 1997 2009 2015	1985 1988 1989 1996 1999 2000 2007 **2010** **2020**	**1982** 1985 1986 1990 1992 1993 1994 2002 2004	1983 1998 2008 **2010** 2016 **2020**

Warm and cold years correspond to normalized PC values greater than 0.75 and less than −0.75, respectively. Years classified as warm or cold in both PC1 and PC3 are shown in bold.

**Table 3:** Annual mean TC genesis frequency (yr^−1^) entering the emergency zone in South Korea for each cluster

	Warm PC1	Cold PC1	Warm PC3	Cold PC3	Total
C1	**0.83-**	**3.33+**			2.18
C2	-	-	**2.33+**	**0.50-**	1.51

Warm and cold years correspond to normalized PC values greater than 0.75 and less than −0.75, respectively. Bold font indicates frequencies that are statistically enhanced or reduced at the 10% significant level, noted by + and −, respectively.

**Figure 5:**
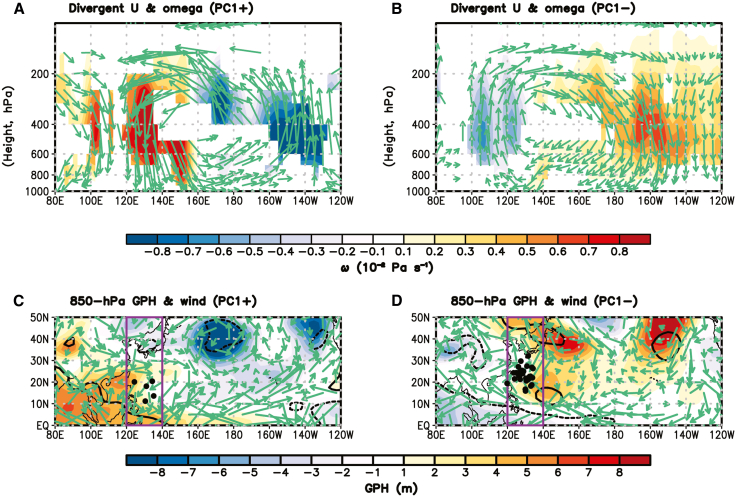
Walker circulation and environmental conditions associated with PC1 (A) Differences in the zonal-vertical profile of divergent zonal wind (vector; m s^−1^) and omega (shaded; 10^−2^ Pa s^−1^), averaged over latitude 5°S-5°N, between the six warm PC1 years and climatology, representing Walker circulation. (B) Same as (A), but for the nine cold PC1 years. (C) Differences in the spatial patterns of 850-hPa geopotential height (GPH, shading; m) and wind (vector; m s^−1^) between the six warm PC1 years and climatology. (D) Same as (C), but for the nine cold PC1 years. Shading in (A) and (B), contours in (C) and (D), and vectors in (A)-(D) indicate statistically significant values at the 10% level, determined using a two-tailed Student’s *t* test. Black dots in (C) and (D) denote the TC genesis locations in C1.

These contrasting statistical relationships are explained by the opposing lower-level large-scale environmental circulation anomalies over the main pathway of C1 TCs, modulated by the EP El Niño and La Niña. In the warm PC1 (i.e., EP El Niño) years, the positive SST anomaly over the EP region weakens the Walker circulation,^25^^,^^26^ inducing an anticyclonic circulation anomaly over the Maritime Continent (Figures 5A and 5C). Simultaneously, a Gill-type response to the SST forcing triggers a cyclonic circulation anomaly over the southeastern WNP^27^ (Figure 5C). This west-east dipolar circulation pattern produces northerly wind anomalies over the main pathway of C1 (120°-140°E; purple box in Figure 5C), acting as a barrier that prevents TCs originating in the Philippine Sea from advancing toward South Korea, leading to a notable decline in C1 frequency (Table 3). In contrast, the cold PC1 (i.e., EP La Niña) years exhibit the opposite circulation pattern compared to the warm PC1 years. The negative SST anomaly over the EP region strengthens the Walker circulation, fostering a cyclonic circulation anomaly over the Maritime Continent (Figures 5B and 5D). Concurrently, the Gill-type response to the SST forcing establishes an anticyclonic circulation anomaly over the southeastern WNP (Figure 5D). These circulation anomalies generate southerly wind anomalies over the main C1 pathway (120°–140°E; purple box in Figure 5D), facilitating the northward movement of TCs formed in the Philippine Sea toward South Korea and thereby significantly increasing C1 frequency (Table 3).

Similarly, we identify nine warm and six cold PC3 years using a normalized PC3 threshold of ±0.75 to investigate the statistical and dynamical relationships between C2 and CP ENSO in more detailed. In the warm PC3 years, an average of 2.33 C2 TCs per year (*p* < 0.1) affect South Korea, whereas in the cold PC3 years, the occurrence of C2 TCs is significantly lower, with only 0.50 years^−1^ (*p* < 0.1) (Table 3).

**Figure 6:**
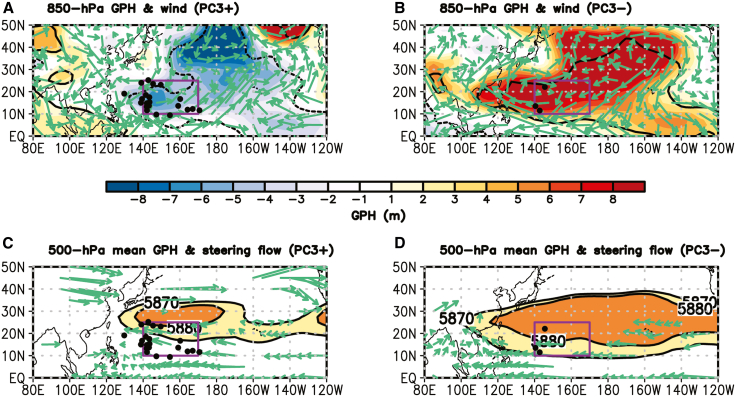
Environmental conditions and variations in western North Pacific subtropical high associated with PC3 (A) Differences in the spatial patterns of 850-hPa GPH (shading; m) and wind (vector; m s^−1^) between the nine warm PC3 years and climatology. (B) Same as (A), but for the six cold PC3 years. (C) 500-hPa mean GPH and environmental steering flow, defined as pressure-weighted, vertically averaged winds between 850- and 300-hPa, for the nine warm PC3 years. (D) Same as (C), but for the six cold PC3 years. Contours in (A) and (B), and vectors (A)-(D), indicate statistically significant values at the 10% level, determined using a two-tailed Student’s *t* test. Black dots in (C) and (D) denote the TC genesis locations in C2.

These reversed statistical results in C2 TC frequency between the warm and cold PC3 years are attributed to differences in lower-level large-scale atmospheric circulation and variations in the WNP subtropical high (WNPSH) associated with CP ENSO phases. During the warm PC3 (i.e., CP El Niño) years, the positive SST anomaly over the CP region acts as a forcing that generates a cyclonic circulation anomaly over the southeastern WNP through a Gill-type response to the SST forcing^27^ (Figure 6A). This anomaly promotes TC genesis in the main development region of C2^2^^,^^11^ (140°–170°E, 10°–25°N) and leads to an eastward shift of the WNPSH edge^28^ (Figure 6C). The eastward-displaced WNPSH edge, together with southeasterly steering flows in the main development region of C2, provides favorable conditions for TCs to follow recurving tracks, thereby increasing C2 frequency (Table 3). In contrast, during the cold PC3 (i.e., CP La Niña) years, the reversed SST anomaly pattern induces an anticyclonic circulation anomaly over the southeastern WNP (Figure 6B). This suppresses TC genesis in the key development region of C2 (140°–170°E, 10°–25°N) and is accompanied by a westward shift of the WNPSH edge, with easterly steering flows along its boundary (Figure 6D). As a result, fewer TCs take a recurved path toward South Korea, leading to a significant decrease in C2 frequency (Table 3).

## Discussion

This study classifies 144 TC tracks that enter emergency zone of South Korea (west of 132°E and north of 28°N) from 1982 to 2020 using the SOM method. The optimal number of clusters is determined through the FDR test, which identifies two distinct TC clusters with different physical characteristics, including genesis location, intensity, and duration (Table 1). Since clustering results can vary depending on the method used, we additionally apply the *k*-means clustering algorithm to the same set of 144 TC tracks, specifying two clusters to match the SOM configuration. However, all tracks are assigned to a single cluster in the k-means approach (not shown), supporting the suitability of the SOM method for capturing distinct TC patterns, particularly when the tracks exhibit nonlinear characteristics and only a small number of clusters is specified.^29^

A key finding of this study is the strong connection between the interannual variability of the two TC clusters and the two types of ENSO (Table 3), a relationship that has not been fully addressed in previous studies.^15^^,^^16^ The interannual indices representing the two ENSO types are derived from EOF analysis (Figure 4). The results show that C1, which consists of TCs developing in the Philippine Sea and moving northward (Figure 3A), is significantly modulated by lower-level environmental conditions induced by EP ENSO. During EP La Niña years, southerly wind anomalies over the main pathway region of C1 (120°–140°E) are generated by a cyclonic circulation anomaly over the Maritime Continent, driven by a strengthened Walker circulation, and an anticyclonic circulation anomaly over the southeast WNP, forced by the EP SST cooling (Figures 5B and 5D). These circulation anomalies enhance the manifestation of C1 (Table 3). In contrast, C2, which includes TCs forming in the southeastern WNP with recurving tracks (Figure 3B), is significantly influenced by lower-level circulation pattern and variation in the WNPSH edge associated with CP ENSO. During CP El Niño years, a cyclonic circulation anomaly over the southeastern WNP, in response to the CP SST warming, increases TC genesis over the main development region of C2 (140°–170°E, 10°–25°N) and shifts the WNPSH edge eastward (Figures 6A and 6C), thereby increasing the occurrence of C2 (Table 3). These relationships are also observed in reverse during EP El Niño and CP La Niña years, demonstrating the robustness of ENSO-TC interaction patterns identified in this study.

**Figure 7:**
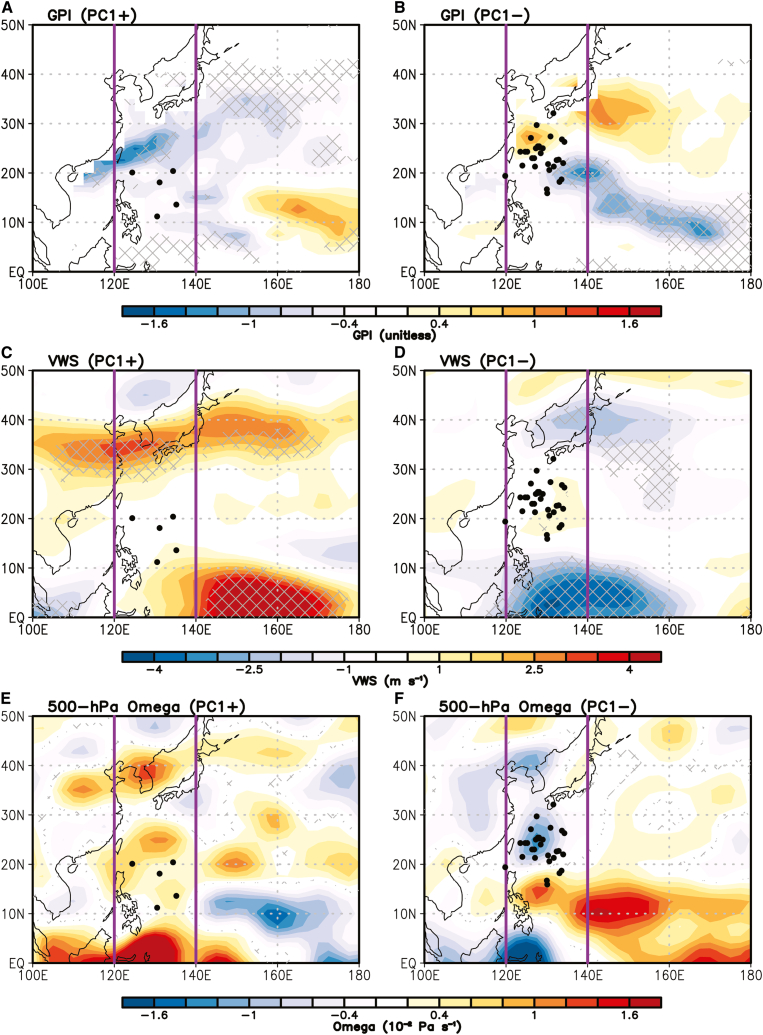
Potential factors that can modulate the TC genesis of C1 Differences in the (A) GPI, (C) VWS, and (E) 500-hPa omega between the six warm PC1 years and climatology. (B), (D), and (F) are same as (A), (C), and (E), but for the nine cold PC1 years. Cross-hatching indicates statistically significant values as the 10% level, determined using a two-tailed Student’s *t* test. Purple box denotes the region of the main pathway C1 (120°–140°E). Black dots denote the TC genesis locations in C1.

Note that the SOM clustering uses TC tracks as input vectors, so the genesis location is only one component of the input and, in practical terms, represents the starting point of the track. Consequently, our primary focus is on how ENSO-induced large-scale environmental circulation anomalies influence the clustered TC tracks. Depending on the ENSO phase and the associated circulation anomalies, these influences may appear solely as changes in track characteristics or as combined changes in both genesis location and track. In this context, considering the main development region of C1 (120°–140°E, 10°–30°N), EP ENSO-related circulation anomalies do not significantly modulate C1 genesis. For example, Gill-type responses to SST forcing generate lower-level circulation anomalies mainly over the eastern WNP (east of 140°E), while Walker circulation-induced circulation anomalies occur west of 120°E, outside the C1 genesis region (Figures 5C and 5D). In addition, potential genesis-related factors, such as genesis potential index (GPI), vertical wind shear (VWS), and mid-level vertical motion (500-hPa omega), also show no statistically significant anomalies there (Figure 7). These results indicate that EP ENSO phases have little direct effect on C1 genesis; instead, their main impact is on TC movement via lower-level wind anomalies. On the other hand, CP ENSO-associated large-scale environmental circulation anomalies influence both the genesis and track of C2 (Figure 6).

**Figure 8:**
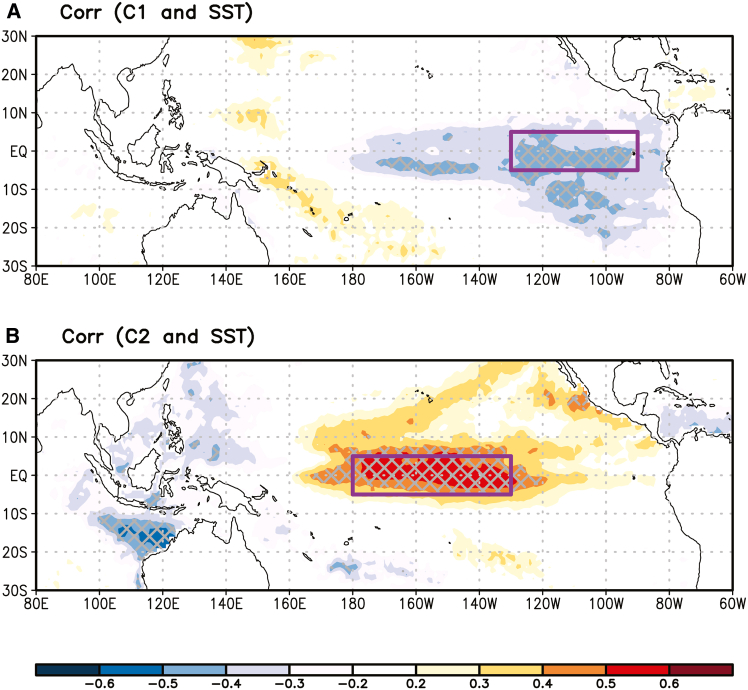
Correlation maps between the two SOM cluster patterns and SST (A) the interannual frequency of C1 and JAS mean SST anomalies during 1982–2020. (B) Same as (A), but for the interannual frequency of C2. Cross-hatching indicates statistically significant correlations at the 10% level, determined using a two-tailed Student’s *t* test. Purple boxes in (A) and (B) denote the regions used for the EP and CP indices.

Similar statistical results can be obtained using area-mean tropical Pacific SST indices instead of EOF-based ENSO indices. To explore this, we examine the correlation maps between the interannual frequency of each cluster and JAS mean SST anomalies. The C1 frequency is negatively correlated with EP SST variation, while the C2 frequency is positively correlated with CP SST variation (Figure 8). Based on these correlation maps, we derive two area-mean ENSO indices: one is the EP index, representing the SST anomaly averaged over (130°W to 95°W, 5°S to 5°N), and the other is the CP index, representing the SST anomaly averaged over (180° to 130°W, 5°S to 5°N). The interannual frequency of C1 and C2 is significantly correlated with the EP index (r = −0.43, *p* < 0.01) and CP index (r = 0.54, *p* < 0.01), respectively. However, while the EP and CP indices are highly correlated (r = 0.69), they are not as distinct as the EOF-based ENSO indices (r = 0.00), which are better separated. This close correlation between the area-mean indices implies a difficulty in obtaining sufficient degrees of freedom to ensure reliable results. In contrast, the EOF-based ENSO indices, with their distinct separation, offer sufficient degrees of freedom and are thus used in this study for more reliable outcomes.

Our findings highlight the potential for developing empirical prediction models for TC frequency affecting South Korea by incorporating the distinct influences of EP and CP ENSO. Given that these two ENSO types exhibit different spatial structures, underlying dynamic mechanisms, temporal evolutions, and teleconnection patterns,^30^^,^^31^ a multiple regression approach accounting for these distinctions could improve seasonal TC forecasts, which will be a subject of future research.

### Limitations of the study

This study classifies TC tracks entering the South Korean emergency zone using the SOM method to capture the nonlinear characteristics of TC trajectories. However, alternative clustering approaches (e.g., K-means or fuzzy C-means) might yield different results. In addition, because ENSO diversity has changed over time, its dominant influence on TC tracks is not stationary. Therefore, the ENSO-TC relationships identified in this study may be specific to the selected analysis period.

## Resource availability

### Lead contact

Further information and requests for resources and reagents should be directed to and will be fulfilled by the lead contact, Jong-Yeon Park (jongyeon.park@jbnu.ac.kr).

### Materials availability

The study did not generate new materials.

### Data and code availability

• Data: All data used in this study are listed in the key resources table.
• Code: The figure codes are publicly available via Zenodo (DOI: https://zenodo.org/records/17180556). Any other custom code not deposited on Zenodo can be made available upon reasonable request from the lead contact.
• Any additional information required to reanalyze the data reported in this paper is available from the lead contact upon request.

## Acknowledgments

This work was supported by the 10.13039/501100003725National Research Foundation of Korea (NRF) under grant numbers RS- RS-2025-00520404 (H.-K.K.), 2024-00461585 (H.-K.K.), RS-2023-00207866 (H.-K.K. and J.-Y.P.), as well as the research funds of 10.13039/501100015499Jeonbuk National University in 2024 (J.-Y.P.).

## Author contributions

Conceptualization, H.-K.K; methodology, H.-K.K. and J.-E.C.; investigation, H.-K.K., J.-Y.P., and J.-E.C.; writing, H.-K.K., J.-Y.P., and J.-E.C.; funding acquisition, H.-K.K. and J.-Y.P.

## Declaration of interests

The authors declare no competing interests.

## STAR★Methods

### Key resources table

**Table:** 

REAGENT or RESOURCE	SOURCE	IDENTIFIER
**Deposited data**		

RSMC TC dataset	Regional Specialized Meteorological Centers Tokyo-Typhoon Center	https://www.jma.go.jp/jma/jma-eng/jma-center/rsmc-hp-pub-eg/besttrack.html
NCEP-NCAR Reanalysis-1 dataset	National Oceanic and Atmospheric Administration	https://psl.noaa.gov/data/gridded/data.ncep.reanalysis.html
OISST version 2 dataset	National Oceanic and Atmospheric Administration	https://psl.noaa.gov/data/gridded/data.noaa.oisst.v2.highres.html
Figure codes	This paper	https://zenodo.org/records/17180556

**Software and algorithms**		

SOM v3.2 (open source)	Kohonen et al.^32^^,^^33^	http://cis.legacy.ics.tkk.fi/hynde/lvq/
IDL	NV5	https://www.nv5geospatialsoftware.com/Products/IDL
GrADS (open source)	Grid Analysis and Display System	http://cola.gmu.edu/grads/

### Experimental model and study participant details

This study did not involve experimental models or study participants.

### Method details

#### Observed and reanalysis datasets

This study utilizes the 6-hourly TC dataset from the Regional Specialized Meteorological Centers Tokyo-Typhoon Center. The dataset includes each TC’s name, date, type, latitude and longitude, central pressure, and maximum sustained wind speed (***V***_***max***_) and is available at https://www.jma.go.jp/jma/jma-eng/jma-center/rsmc-hp-pub-eg/besttrack.html. TCs are typically classified based on ***V***_***max***_ into three categories: tropical depressions (***V***_***max***_ < 17 m s^-1^), tropical storms (17 m s^-1^ ≤ ***V***_***max***_ < 34 m s^-1^), and typhoons (***V***_***max***_ ≥ 34 m s^-1^). TCs in this study refer to tropical storms and typhoons.

The reliability of the TC dataset, demonstrating that geostationary satellite observations have been incorporated since 1982 is accessed.^34^ Accordingly, this study focuses on the period from 1982 to 2020. The analysis season is defined as July to September (JAS), as approximately 85% of TCs affecting South Korea enter the emergency zone during this period (Figure 1B). Therefore, unless otherwise stated, all analyses and results are based on JAS averages.

Following the operational classification of the Korea Meteorological Administration (KMA),^7^ TCs affecting South Korea are defined as those whose centers enter the emergency zone (west of 132°E and north of 28°N; purple box in Figure 1A). Although not all make landfall, their size and proximity pose significant threats. As a result, 144 TCs are selected.

To investigate the large-scale environment conditions associated with cluster patterns, we use daily mean atmospheric datasets from National Centers for Environmental Prediction-National Center for Atmospheric Research reanalysis version 1 with a horizontal resolution of 2.5° by 2.5°.^35^ Additionally, we employ high-resolution daily mean SST data from the National Oceanic and Atmospheric Administration optimum interpolation SST version 2,^36^ interpolated from 0.25° by 0.25° to 1° by 1°.

#### Interpolation of TC track

Both SOM and other clustering methods require input vectors of equal length; however, TC tracks typically have different lengths. To address this, previous studies have proposed an interpolation method to standardize TC tracks.^5^^,^^37^ In this study, given that the mean duration of TCs in the entire WNP is approximately five days, each TC track is linearly interpolated into 20 segments (i.e., four steps per day at 6-hour intervals over five days). The interpolated longitude and latitude positions of each TC track are then structured as input vectors for the SOM, represented as follows:

(Equation 1) $$\begin{array}{c} V_{i}=\left[{lon}_{1},{lon}_{2},\ldots .,{lon}_{21},{lat}_{1},{lat}_{2},\ldots .,{lat}_{21}\right] \end{array}$$

where ***V***_***i***_ is the vector for the *i*-th TC that enters the emergency zone in South Korea, and *lon* and *lat* represent the interpolated longitude and latitude, respectively.

#### Self-organizing map

The SOM is an unsupervised learning algorithm based on artificial neural networks that enables self-organizing and clustering of input data without external supervision.^5^^,^^17^^,^^18^^,^^32^^,^^33^^,^^38^ SOM projects continuous input data of arbitrary dimensionality onto low-dimensional and discrete output layers through training and mapping processes. The training process consists of three main steps:

1. Initialization – A set of weight vectors, corresponding to the number of clusters k, is randomly initialized.
2. Competition – The weight vector with the minimum Euclidean distance to the input vector is identified as the winner.
3. Adaptation – The winner’s weight vector and its neighboring weight vectors, determined by a user-defined neighborhood radius and topological neighborhood function, are updated iteratively using the following rule:

(Equation 2) $$\begin{array}{c} W_{k}\left(t+1\right)=W_{k}\left(t\right)+\alpha \left(t\right)nh\left(t\right)\left[V\left(t\right)-W_{k}\left(t\right)\right] \end{array}$$

where ***V*** is the input vector, ***W***_***k***_ is the weight vector of the *k*-th cluster. α is the learning rate, which typically decreases monotonically during the user-defined iterations *t*, and *nh* is the topological neighborhood function. In this application, a Gaussian neighborhood function is used to ensure smooth adaptation. The learning rate *α* is set to 0.05, and the total number of iterations *t* is one hundred thousand.

The adaptation process is a key distinguishing feature of SOM. Unlike conventional clustering methods, e.g., K-means, SOM updates not only the winner weight vector but also its neighboring weight vectors, which contributes to SOM’s inherently nonlinear nature.^5^^,^^17^^,^^18^^,^^38^ Additionally, the weight vectors are arrayed in an ordered structure, facilitating easier interpretation and diagnostics of the results.^5^^,^^18^

After the training phase, the mapping process is performed to assign each input vector to a specific cluster based on the set of final weight vectors. This assignment is determined by minimizing the Euclidean distance between the input vector and the final weight vectors. For more detailed information on SOM, refer to Kohonen.^32^^,^^33^ The SOM-PAK version 3.2 is available at http://cis.legacy.ics.tkk.fi/hynde/lvq/.

Several clustering algorithms have been applied in previous studies to classify TC tracks, including K-means^39^^,^^40^ and Fuzzy C-means clustering methods.^37^ K-means is a partitioning method that minimizes the within-cluster sum of squares but can be sensitive to the initial choice of centroids. Fuzzy C-means allows each input vector to belong to multiple clusters with varying membership degrees,^5^ offering flexibility for overlapping patterns but requiring the specification of a fuzziness parameter. In contrast, the SOM preserves the topological relationships of the input vectors while capturing nonlinear features of TC tracks, making it particularly well-suited for identifying coherent track patterns.

#### False discovery rate

The physical and statistical characteristics of clustered patterns can vary significantly depending on the chosen number of clusters; therefore, determining the optimal cluster number is a critical step in any clustering method. Although various heuristic methods for selecting the number of clusters have been proposed in previous studies,^37^ determining this number remains challenging. Here, we introduce the FDR, which is one of the global significance tests, to identify the maximum number of statistically distinguishable clusters.^5^^,^^18^^,^^41^^,^^42^^,^^43^ In brief, if the number of clusters is *k*, all pairs of clusters (i.e., *k*C2) are statistically distinguishable from each other, while if the number of clusters is *k*+1, at least one pair of clusters is statistically indistinguishable from each other, *k* is selected as the optimal cluster number.

To determine whether a pair of clusters—specifically, SOM cluster pattern *i* and *j*—are statistically distinguishable, we first calculate the t-value using the two-tailed Student’s t-test for different means and then convert the *t*-value to a *p*-value at each point. In this study, since the input vector for SOM is structured as the interpolated longitude and latitude positions of 42 points, the null hypothesis states that the local (i.e., each position) mean of the interpolated track is the same in both clusters. Finally, the FDR test is performed as follows:

(Equation 3) $$\begin{array}{c} P_{FDR}=\max_{1\leq m\leq M}\left[\;p\left(m\right):p\left(m\right)\leq q\cdot \frac{m}{M}\right] \end{array}$$

where ***p***(***m***) denotes the *p*-values sorted in ascending order, and m varies from 1 to *M*, where *M* is the total number of interpolated positions (i.e., 42). The sorted p-values are compared to *q·m/M*, which represents the global significance level, with *q* set to 0.01. If *q* is used directly in Equation 3 instead of *q·m/M*, the test corresponds to a sequential local t-test. By applying the global significance level, the FDR test becomes more stringent. Thus, ***P***_***FDR***_ represents the probability that a true null hypothesis is incorrectly rejected.^42^ If none of the interpolated positions satisfy the condition in Equation 3 for a given pair of clusters, the two clusters are considered statistically indistinguishable. In contrast, if at least one interpolated position satisfies the condition in Equation 3, the null hypothesis is rejected, indicating that the two clusters are statistically distinguishable.

### Quantification and statistical analysis

All statistical analyses were performed using two-tailed Student’s *t*-tests. Statistical significance was defined as the 10% level unless otherwise stated. Figures were created using IDL version 8.1 (Figures 1, 2, and 3) and Grads version 2.1 (Figures 4, 5, 6, 7, and 8).
